# Identification of suitable reference genes for studies of *Syringa pinnatifolia* Hemsl.

**DOI:** 10.1002/2211-5463.13097

**Published:** 2021-02-26

**Authors:** Jiaqi Gao, Juan Liu, Chao Jiang, Suyile Chen, Luqi Huang

**Affiliations:** ^1^ National Resource Center for Chinese Materia Medica China Academy of Chinese Medical Sciences Beijing China; ^2^ School of Pharmacy Jiangsu University Zhenjiang China; ^3^ Alashan Mongolian Hospital Alashan East Banner of Alashan Inner Mongolia China

**Keywords:** gene expression, qRT‐PCR, reference gene, *Syringa pinnatifolia* Hemsl.

## Abstract

*Syringa pinnatifolia* Hemsl. (Oleaceae) is a species of shrub with a limited distribution in China. Several chemical compounds with pharmacological effects have been isolated from *S*. *pinnatifolia*, including new lignans and sesquiterpenes. Studies of gene expression in this species require the identification of suitable reference genes that are stably expressed under different conditions and in different tissues. To identify candidate reference genes, here we used the geNorm, NormFinder, and BestKeeper algorithms to analyze the stability of 12 candidate genes. The geometric mean of the rankings generated with these algorithms was used to obtain a comprehensive ranking. *TBP* and *PP2A* were found to be appropriate reference genes for calli treated with different external stimuli, and *TIP41* and *TBP* were found to be appropriate reference genes in differentiated tissues. When calli and differentiated tissues were considered together, *TBP* and *TIP41* were found to be the most reliable reference genes. The selected genes were validated by analysis of *HMGR* expression in calli and differentiated tissues. This study is the first to screen candidate reference genes in the genus *Syringa* and could help guide future molecular studies in this genus.

## Graphical abstract

Appropriate and stable reference genes are important for normalization of gene expression. Here, we estimated the stability of 12 candidate reference genes in *Syringa pinnatifolia*. This study is the first to screen candidate reference genes in the genus *Syringa* and could help guide future molecular studies in this genus.

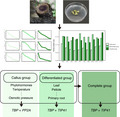

**Abbreviations**
ABA: abscisic acid
ACC: 1‐aminocyclopropane‐1‐carboxylic acid
*ACT*: actin
*bHLH*: basic helix‐loop‐helix transcription factor
CK: untreated control callus
*C*_t_: cycle threshold
CV: percentage covariance
CWS: cultivated woody stem
*EF1α*: elongation factor 1‐alpha
ET: ethylene
FR: fibrous root
GA_3_: gibberellin A_3_
*Helicase*: ATP‐dependent RNA helicase DExH7
*HMGR*: 3‐hydroxy‐f3‐methylglutaryl‐coenzyme A reductase
JAs: jasmonates
MeJA: methyl jasmonate
*PP2A*: protein phosphatase 2A
PR: primary root
PT: petiole
*PTB*: polypyrimidine tract‐binding protein
qRT‐PCR: quantitative reverse transcription–polymerase chain reaction
RH: relative humidity
SA: salicylic acid
SD: standard deviation
*TBP*: TATA box‐binding protein
*TIP41*: TIP41‐like protein
*TUB*: tubulin beta chain‐like protein
*UBCE2*: ubiquitin‐conjugating enzyme 2
*UPL7*: E3 ubiquitin–protein ligase 7
WWS: wild woody stem
*YLS8*: thioredoxin‐like protein YLS8
YS: young stem

*Syringa pinnatifolia* Hemsl. (Oleaceae) is a species of shrub with a limited distribution in China [1]. Its peeled stems, roots, and twigs, called *shanchenxiang* in Chinese, are important in traditional Mongolian medicine [2], where they are used to treat palpitations, angina pectoris, myocardial ischemia, and other cardiovascular diseases [3].

Researchers exploring the pharmacological properties of *S*. *pinnatifolia* have isolated and identified a variety of chemical compounds, including new lignans [4, 5] and sesquiterpenes [6, 7, 8, 9], as well as compounds known from other plants [10, 11]. A number of these compounds, acquired from different tissues, exhibit pharmacological effects, including antibacterial [6, 10], anti‐inflammatory [10, 12], antioxidant [4, 8], antitumor [13], and cardioprotective [10] effects. The main compounds responsible for the pharmacological properties of *S. pinnatifolia* are the lignans and terpenes, with the sesquiterpenes showing the strongest effects. Accordingly, it may be useful to determine the biological mechanism governing the distribution of these components within the plant.

The highest‐quality *shanchenxiang* is derived from mature wild *S. pinnatifolia,* whose stems are full of purple tyloses accumulated over years [3]. To our knowledge, the wild plants of *S. pinnatifolia* have a higher concentration of secondary metabolites, especially lignans and sesquiterpenes, than cultivated or young ones [7, 13, 14, 15, 16]. It is known that the anatomical, physiological, and biochemical characteristics of a plant may change with age [17]. Because biotic and abiotic stresses play essential roles in the production of secondary metabolites, multiple elicitors or signal molecules, such as jasmonates (JAs), salicylic acid (SA), and ethylene (ET), have been used to simulate environmental stresses to promote the accumulation of these compounds [18]. JAs, including jasmonic acid and its derivatives, are phytohormones involved in plant growth, development, and defensive reactions [19]. In addition, JAs can directly regulate the biosynthesis of various secondary products, including terpenoids, flavonoids, alkaloids, and phenylpropanoids [18]. SA could induce the systematic acquired resistance in plant–pathogen interaction, and increased SA concentration is linked to the production of many metabolites [18]. Interestingly, previous studies have shown that the SA and JA pathways can interact in a complex manner [20, 21]. ET is another important stress phytohormone, regulating a variety of plant processes including secondary metabolism [18]. Furthermore, ET is responsible for the formation of tyloses [22], which are a key marker of high‐quality *shanchenxiang*. Additional important factors influencing plant development include heat, cold, and osmotic stresses, which have an impact on the regulation of plant hormones [23]. For instance, osmotic pressure can regulate morphological and developmental changes, ion transport, and metabolism, either directly or through secondary signals [24], and there have been some studies focusing on its role in the regulation of plant hormones such as the auxin indole‐3‐acetic acid [25]. However, little is known about the molecular mechanism of secondary metabolism regulation in *S. pinnatifolia*.

Since the concept of the transcriptome and its applications was first described in a study on yeast [26], transcriptomics has been used in many studies of traditional medicinal plants [27, 28]. The transcriptomics approach is often used for quantitative analysis of gene expression; for decades, the most effective tool for such studies has been the quantitative reverse transcription–polymerase chain reaction (qRT‐PCR), with various companies supplying products to facilitate the technique, such as TaqMan, LightCycler, LUX, molecular beacons, and SYBR Green [29]. qRT‐PCR has many advantages, such as accuracy, broad dynamic range, and sensitivity [30, 31]; however, this accuracy can lead to the amplification of errors. Therefore, it is necessary to select a housekeeping gene—a gene that is stably expressed under different conditions and in different tissues—as an internal control; this is called the reference gene [32]. However, recent studies have shown that the expression of housekeeping genes can vary depending on experimental conditions, which means there is no universal reference gene that can be used under every condition [33]. For this reason, it is essential to determine the appropriate reference genes to suit specific tests.

In this study, 12 candidate reference genes selected from the transcriptome dataset of *S. pinnatifolia* were evaluated to determine the most appropriate genes to use as references for qRT‐PCR data normalization. Data were obtained from different plant samples, some of which were subjected to various treatments, and qRT‐PCR results were analyzed using three normalization algorithms: geNorm [34], NormFinder [35], and BestKeeper [36].

## Materials and methods

### Plant materials

**Fig. 1:**
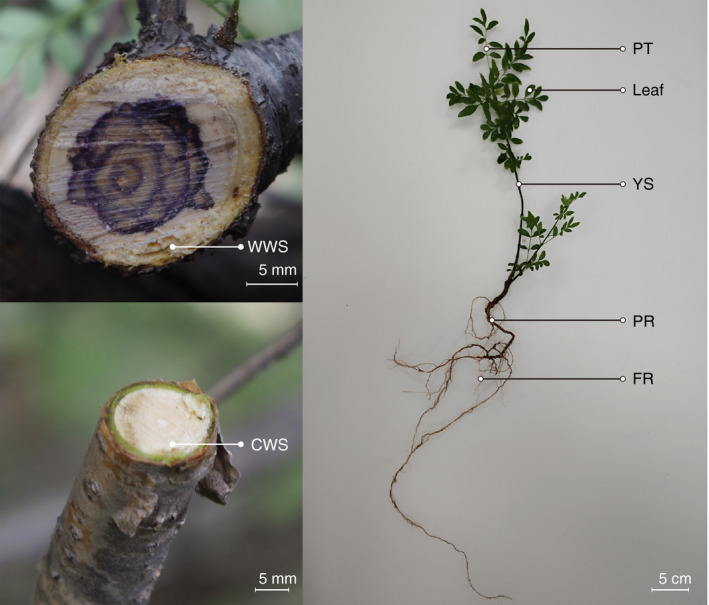
*Syringa pinnatifolia* tissues used in qRT‐PCR. WWS, wild woody stem; CWS, cultivated woody stem; PT, petiole; YS, young stem; PR, primary root; FR, fibrous root. The lengths of the scale bars in the figures of WWS and CWS are both 5 mm. The scale bar in the figure of the whole plant is 5 cm long.

Wild woody stems of *S*. *pinnatifolia* were collected from Xiazi Gully (N38°29′32″, E105°50′2″, location‐1; N38°29′29″, E105°49′54″, location‐2; N38°29′42″, E105°50′3″, location‐3; N38°29′43″, E105°50′5″, location‐4), Mount Helan Nature Reserve, Inner Mongolia, China (Fig. 1). Cultivated woody stems of *S. pinnatifolia* were collected from a cultivation center at Alxa League Traditional Mongolian Medicine Hospital (N38°42′43″, E105°33′2″, location‐1 to location‐4), Inner Mongolia, China. For samples from different tissues, leaf, petiole, young stem, primary root, and fibrous root were collected from 3‐year‐old seedlings of *S. pinnatifolia* grown in a greenhouse at 20–25 °C and 35% relative humidity (RH) at the National Resource Center for Chinese Materia Medica, China Academy of Chinese Medical Sciences, Beijing, China.

For callus culture, the leaves of *S. pinnatifolia* were collected and surface sterilized with mercuric bichloride and 75% ethanol. After being washed 10 times with sterile water, the leaf pieces (1 cm × 1 cm) were plated on the Murashige and Skoog (MS) medium with 1.0 μg·mL^−1^ naphthalene‐1‐acetic acid (NAA) and 1.2 μg·mL^−1^ 6‐benzylaminopurine (6‐BA) for callus induction. Induced calli were subcultured every month to fresh maintenance medium (MS medium with 1.0 μg·mL^−1^ NAA and 1.2 μg·mL^−1^ 6‐BA). For stress and hormone treatments, calli were transferred to MS liquid solutions supplemented with 400 mm mannitol, 50 mm H_2_O_2_, 300 mm NaCl, 100 μm abscisic acid (ABA), 100 μm methyl jasmonate (MeJA), 100 μm salicylic acid (SA), 50 μm brassinolide, 50 μm zeatin, 50 μm 1‐aminocyclopropane‐1‐carboxylic acid (ACC), 50 μm gibberellin A_3_ (GA_3_), 50 μm ethephon and incubated for 6 h. The mock‐treated calli, incubated in MS liquid medium with 50 or 100 μm ethanol, were served as the controls, respectively, for the hormones were dissolved in ethanol. To further detect the temperature effects on *S. pinnatifolia*, the calli were treated at different temperatures (42 °C and 4 °C), and the ones (CK) cultivated at 25 °C were used as control.

All the callus samples, regardless of treatment, were considered together as the ‘callus group’; the samples of differentiated tissues were considered together as the ‘differentiated group’; the ‘complete group’ was the superset containing all of the samples in ‘callus group’ and ‘differentiated group’. All the samples were immediately frozen in liquid nitrogen and stored at −80 °C until subsequent use. Each sample in ‘callus group’ or ‘differentiated group’ used for the subsequent experiments contains four biological replicates.

### RNA extraction and complementary DNA synthesis

Total RNA was extracted from the *S. pinnatifolia* samples using TRIzol reagent (Thermo Fisher Scientific, Waltham, MA, USA) according to the manufacturer's instructions. Each sample used in RNA extraction was approximately 200 mg. DNA was removed using gDNA eraser (Takara Bio, Kusatsu, Japan). RNA integrity was checked by agarose gel electrophoresis. The concentration and purity of extracted RNA were determined by using a NanoDrop 2000 spectrophotometer (Thermo Fisher Scientific). RNA samples were considered to have ideal optical density (OD) ratios at the following values: OD_260_/OD_280_, between 1.8 and 2; OD_260_/OD_230_, greater than 2. Subsequently, 0.5 μg RNA per sample was reverse‐transcribed into single‐stranded complementary DNA (cDNA) using a cDNA synthesis kit (Takara Bio) following the manufacturer's instructions.

### Selection of reference genes and primer design

A total of 12 candidate reference genes (Table S1) were selected from the *S. pinnatifolia* transcriptome dataset (unpublished), namely *ACT* (actin), *bHLH* (basic helix‐loop‐helix transcription factor), *EF1α* (elongation factor 1‐alpha), *Helicase* (ATP‐dependent RNA helicase DExH7), *PP2A* (protein phosphatase 2A), *PTB* (polypyrimidine tract‐binding protein), *TBP* (TATA box‐binding protein), *TIP41* (TIP41‐like protein), *TUB* (tubulin beta chain‐like protein), *UBCE2* (ubiquitin‐conjugating enzyme 2), *UPL7* (E3 ubiquitin–protein ligase 7), and *YLS8* (thioredoxin‐like protein YLS8). The conserved domains of these candidate reference genes were identified using the blastn database (National Center for Biotechnology Information, Bethesda, MD, USA). Primer pairs used for qRT‐PCR were designed based on these conserved domains using primer premier 5 (Premier Biosoft, San Francisco, CA, USA). The primer pairs were synthesized by a commercial supplier (Sangon, Beijing, China) and tested in regular PCR, and the products were run on 2% agarose gel.

### qRT‐PCR

qRT‐PCR was carried out in 96‐well plates with a LightCycler480 real‐time PCR system (Roche Molecular Systems, Mannheim, Germany). The reaction mixture contained 3 μL of ultrapure water, 5 μL of TB Green Premix Ex Taq II (TaKaRa Bio), 1 μL of cDNA, 0.5 μL of forward primer (10 μm), and 0.5 μL of reverse primer (10 μm). The program was set to run for 1 min at 94 °C, 40 cycles of 10 s at 94 °C, and 34 s at 60 °C. Melt curves were obtained by heating the sample from 60 °C to 95 °C at a rate of 1.0 °C·s^−1^. Three technical replicates were run for each sample. The amplification efficiency (*E*) and correlation coefficients (*R* ^2^) were calculated using a standard curve based on fivefold serial dilution of a mixture of the synthesized cDNA over 10 dilution points, starting from a concentration of 300 ng·µL^−1^. Negative controls with water instead of cDNA were included for each target gene under assay.

### Analysis of gene expression stability

Three statistical tools, geNorm [34], NormFinder [35], and BestKeeper [36], were used to evaluate gene expression stability in the experimental samples. The geNorm program calculates the average expression stability measurement (*M*) value according to the pairwise variation between two sequences, eliminating the genes that show the worst expression stability in a stepwise manner. NormFinder calculates the stability value based on variance analysis; this avoids the limitations of geNorm, which cannot discriminate between coregulated genes. BestKeeper evaluates expression stability by calculating standard deviation (SD) and percentage covariance (CV). BestKeeper and geNorm are both based on pairwise comparison so that they have the same vulnerabilities regarding coregulated genes. The comprehensive ranking order of gene stability was determined using the geometric mean of the ranks from geNorm, NormFinder, and BestKeeper.

### Validation of reference genes

To validate the selected reference genes, the expression of *HMGR* (3‐hydroxy‐3‐methylglutaryl‐coenzyme A reductase; GenBank accession number MW149516), which codes for the rate‐limiting enzyme in the mevalonate pathway involved in terpene biosynthesis [37], was analyzed using the top two genes in the comprehensive ranking order as references, both separately and in combination. For comparison, the lowest‐ranking genes in each group were also used. The $2^{‐\Delta \Delta C_{t}}$ method [38] was used to calculate the level of *HMGR* expression in each group.

## Results

### Verification of primers

The primer pairs used for the 12 selected candidate reference genes are given in Table 1. Their PCR efficiencies ranged from 90.39% to 105.49%, with appropriate correlation coefficients. Gel electrophoresis produced clear single bands without impurities (Fig. S1A), with amplicon sizes ranging from 93 to 258 bp, which were consistent with our purpose (Table 1). Melt curves for all primers showed a single peak (Fig. S1B), indicating that they were specific for the selected genes and thus suitable for qRT‐PCR.

**Table 1:** Candidate reference genes and primer sequences.

Gene symbol	Homologous locus accession^a^	Description	Primer sequences forward/reverse (5′–3′)	Tm (°C)	Amplicon size (bp)	PCR efficiency (%)	*R* ^2^
*ACT*	XM_023027148.1	Actin protein	GGCTGTCTTCCCCAGTATCG	58.20	258	92.16	0.9990
			GCCTTGGGGTTAAGAGGAGC	58.81			
*bHLH*	XM_023042644.1	Basic helix‐loop‐helix transcription factor	TCTTGGGAGATGCCATCACTT	55.99	225	95.34	0.9984
			GGAATGCCTGGATGACCCT	57.63			
*EF1α*	XM_028228415.1	Elongation factor 1‐alpha	CCCTGACAAGATCCCGTTTG	56.20	172	90.39	0.9976
			GACATCCTGAAGTGGGAGACG	57.87			
*Helicase*	XM_017766571.1	ATP‐dependent RNA helicase DExH7	GATGAGCGATGTGAACCGTCT	57.41	156	103.85	0.9979
			AACACCAGCCAAGTCTTTATTCC	55.08			
*PP2A*	XM_022998606.1	Protein phosphatase 2A	ATTGGATTACAGATGAGGGAGAATG	53.90	110	98.52	0.9998
			AACAGGCCACAAGCAGAAACT	57.32			
*PTB*	XM_023023790.1	Polypyrimidine tract‐binding protein	CTTTCGGCAAGATCGTCAATAC	53.73	255	96.46	0.9979
			CATCACCAGCTTCAACTCCCT	57.32			
*TBP*	XM_022993373.1	TATA box‐binding protein	AATTGGCTGCTCGGAAGTATG	55.26	159	95.91	0.9998
			CGTAACTTGAAAAGGCACCATG	54.41			
*TIP41*	XM_023020543.1	TIP41‐like protein	TGGACGGAGTGCTTATGAGATT	55.56	193	99.64	0.9982
			ATGATGATTGGGAGCCTTTTG	52.78			
*TUB*	XM_024307833.1	Tubulin beta chain‐like	ACAGGTAGGAACAAGGGTGAGG	58.95	189	96.30	0.9988
			AAAGTTATCGGGTCGGAAGATC	54.18			
*UBCE2*	XM_023013227.1	Ubiquitin‐conjugating enzyme 2	CCCCACTTCACCCTCACATT	57.55	213	95.96	0.9993
			GGAACCACCATCTCGTCTCCT	59.15			
*UPL7*	XM_023033190.1	E3 ubiquitin–protein ligase 7	CGGAAAGTTGCCTCAATGGT	55.76	165	105.49	0.9982
			AGGTGAAAGCCACAAAGTAGGAG	56.95			
*YLS8*	XM_023025802.1	Thioredoxin‐like protein YLS8	GGGCACTGAAGGATAAACAGGA	56.96	93	97.33	0.9995
			CTTTCGGAGCAATAACTAGACCAC	55.32			

^a^ The homologous locus accession is the accession numbers of homologous locus of *Olea europaea*.

### Expression profiles of candidate reference genes

**Fig. 2:**
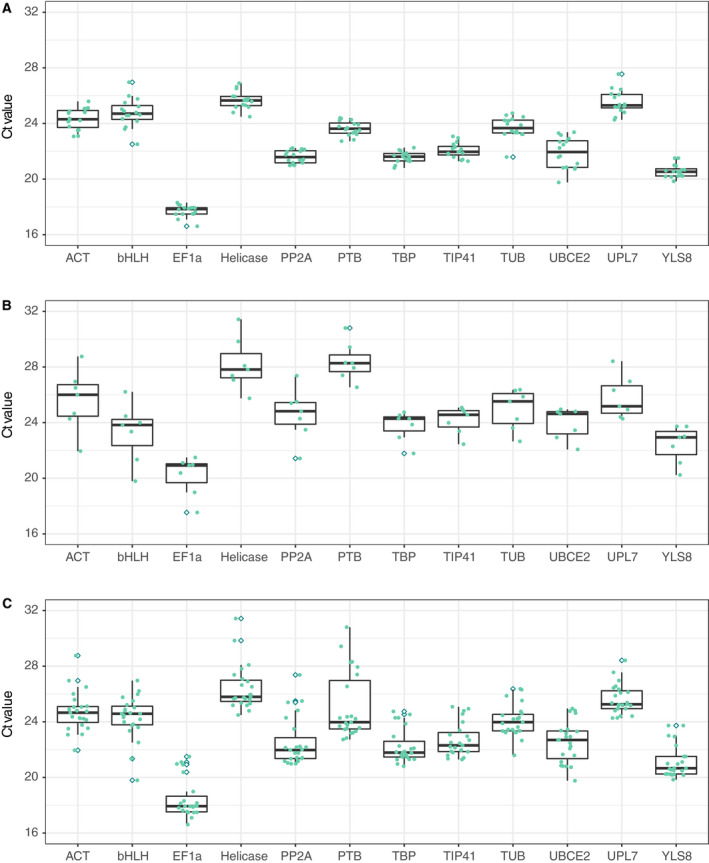
Expression profiles of candidate genes. (A) Expression of candidate genes in the callus group. (B) Expression of candidate genes in the differentiated group. (C) Expression of candidate genes in complete group.

In qRT‐PCR analysis, a high cycle threshold (*C*_t_) value indicates low gene expression, while a low *C*_t_ value suggests high gene expression. In our study, *C*_t_ values for all genes remained within the range of 15–35 (Fig. 2, Data S1). It was clear that the numerical distributions in the callus and differentiated groups were similar (Fig. 2A,B). However, there were also some differences; for example, most genes showed less variation in expression levels in the callus group (Fig. 2A) than in the differentiated group (Fig. 2B). Additionally, the expression levels of most genes were higher in the callus group (Fig. 2A) than in the differentiated group (Fig. 2B), except for *bHLH*. The genes exhibiting the lowest levels of expression were *Helicase* and *UPL7* in the callus group and *Helicase* and *PTB* in the differentiated group. For all samples considered together, *EF1α* had the lowest median *C*_t_ value and *Helicase* had the highest (Fig. 2C), while *PTB* and *UBCE2* had the highest variation in expression levels.

### Stability analyses of candidate reference genes

**Fig. 3:**
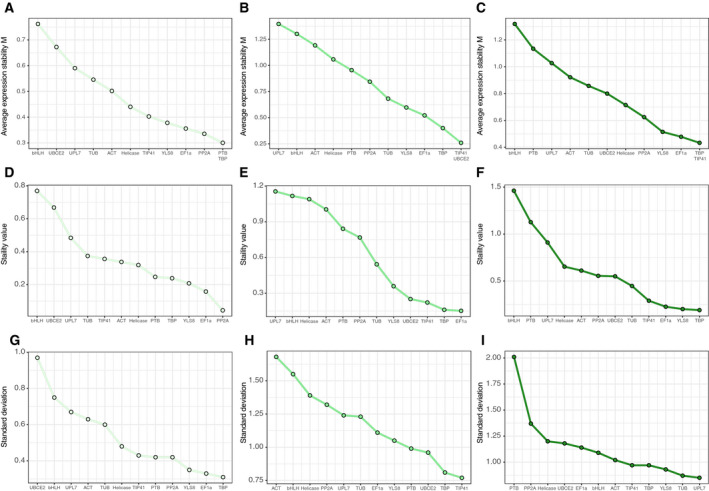
Stability analyses of candidate genes. (A–C) geNorm analysis of genes in calli, differentiated tissues, and complete group. (D–F) NormFinder analysis of genes in calli, differentiated tissues, and complete group. (G–I) BestKeeper analysis of genes in calli, differentiated tissues, and complete group.

The geNorm program evaluates the stability of reference gene expression by calculating the *M* value [34] with a high *M* value suggesting poor stability. According to *M* value, *PTB* and *TBP* were the two most stable genes in the callus group, while *bHLH* and *UBCE2* were the two most unstable (Fig. 3A). In contrast, the two most stable genes in the differentiated group were *TIP41* and *UBCE2* (Fig. 3B), despite the latter being one of the most unstable in the callus group. For both groups taken together, the most stable genes were *TBP* and *TIP41* (Fig. 3C).

**Fig. 4:**
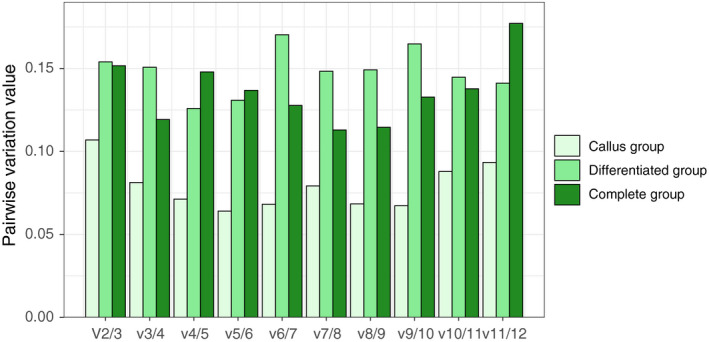
Minimum number of reference genes for reliable normalization. The *V_n_*/*V_n_*_ + 1_ value on the horizontal axis corresponds to the pairwise variation value. When the ratio is less than 0.15, the appropriate gene number is *n*; when the corresponding ratio is larger than 0.15, the number would be *n* + 1.

The geNorm program uses pairwise variation (*V_n_*/*V_n_*_ + 1_) to estimate the optimal number of reference genes, where a ratio of less than 0.15 means *n* is the recommended number [34]. As shown in Fig. 4, all pairwise variation ratios in the callus group were less than 0.15, suggesting that two reference genes would be necessary in this group, while for the differentiated group, the number was larger at four. For both groups taken together, pairwise variation ratios suggested the optimal number of reference genes to be three.

NormFinder generates a stability value based on intragroup and intergroup variation in expression [35], with a high value indicating low stability, as with the *M* value generated by geNorm. In our analysis, NormFinder found that the most stable gene in the callus group was *PP2A*, whereas the most stable gene in the differentiated group was *EF1α* (Fig. 3D,E). For the complete group, *TBP* was the most stable, with the lowest NormFinder stability value (0.190) (Fig. 3F).

BestKeeper estimates the stability of reference genes by calculating SD and percentage CV [36]. Genes with an SD less than 1 are considered stable; by this measure, the number of stable genes in the callus group was much larger than that in the differentiated group (Fig. 3G,H) [36]. For the complete group, there were five candidate reference genes with an SD less than 1, with the most stable being *UPL7* (Fig. 3I).

To obtain a comprehensive overall ranking, we calculated the geometric mean of the stability rankings generated by geNorm, NormFinder, and BestKeeper, which differed on account of their calculation methods (Table 2). After the reranking, *TIP41* appeared to be the best candidate reference gene in the tissue group, while *TBP* was the best in the callus group and the complete group. Although *bHLH* was the most unstable gene in both the callus and differentiated groups, *PTB* was the most unstable when both groups were taken together.

**Table 2:** Ranking of the candidate reference genes analyzed through different algorithms and the overall rank.

Group	Callus group				Differentiated group				Complete group			
Rank	geNorm	NormFinder	BestKeeper	Overall	geNorm	NormFinder	BestKeeper	Overall	geNorm	NormFinder	BestKeeper	Overall
1	*TBP/PTB*	*PP2A*	*TBP*	*TBP*	*TIP41/UBCE2*	*EF1α*	*TIP41*	*TIP41*	*TBP/TIP41*	*TBP*	*UPL7*	*TBP*
2	Null	*EF1α*	*EF1α*	*PP2A*	Null	*TBP*	*TBP*	*TBP*	Null	*YLS8*	*TUB*	*TIP41*
3	*PP2A*	*YLS8*	*YLS8*	*EF1α*	*TBP*	*TIP41*	*UBCE2*	*UBCE2*	*EF1α*	*EF1α*	*YLS8*	*YLS8*
4	*EF1α*	*TBP*	*PP2A*	*PTB*	*EF1α*	*UBCE2*	*PTB*	*EF1α*	*YLS8*	*TIP41*	*TBP*	*EF1α*
5	*YLS8*	*PTB*	*PTB*	*YLS8*	*YLS8*	*YLS8*	*YLS8*	*YLS8*	*PP2A*	*TUB*	*TIP41*	*TUB*
6	*TIP41*	*Helicase*	*TIP41*	*TIP41*	*TUB*	*TUB*	*EF1α*	*TUB*	*Helicase*	*UBCE2*	*ACT*	*UPL7*
7	*Helicase*	*ACT*	*Helicase*	*Helicase*	*PP2A*	*PP2A*	*TUB*	*PTB*	*UBCE2*	*PP2A*	*bHLH*	*UBCE2*
8	*ACT*	*TIP41*	*TUB*	*ACT*	*PTB*	*PTB*	*UPL7*	*PP2A*	*TUB*	*ACT*	*EF1α*	*PP2A*
9	*TUB*	*TUB*	*ACT*	*TUB*	*Helicase*	*ACT*	*PP2A*	*Helicase*	*ACT*	*Helicase*	*UBCE2*	*ACT*
10	*UPL7*	*UPL7*	*UPL7*	*UPL7*	*ACT*	*Helicase*	*Helicase*	*ACT*	*UPL7*	*UPL7*	*Helicase*	*Helicase*
11	*UBCE2*	*UBCE2*	*bHLH*	*UBCE2*	*bHLH*	*bHLH*	*bHLH*	*UPL7*	*PTB*	*PTB*	*PP2A*	*bHLH*
12	*bHLH*	*bHLH*	*UBCE2*	*bHLH*	*UPL7*	*UPL7*	*ACT*	*bHLH*	*bHLH*	*bHLH*	*PTB*	*PTB*

### Validation of the selected reference genes

**Fig. 5:**
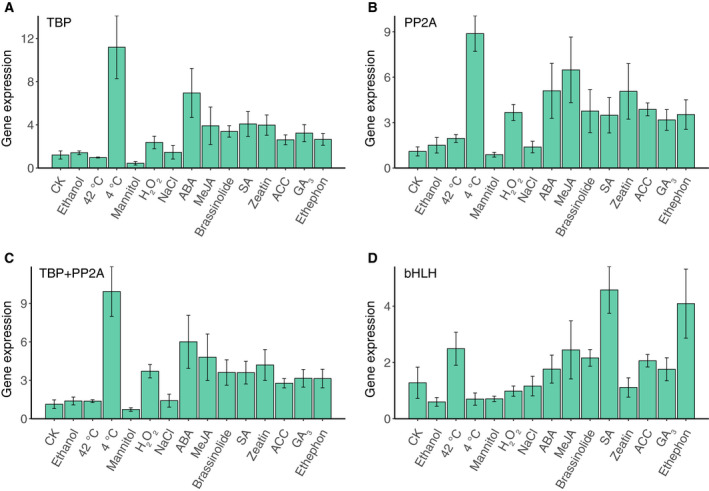
Relative expression levels of *HMGR* normalized using the reference genes in the callus group. (A, B) Relative expression levels of *HMGR* normalized using the top two ranked genes. (C) Relative expression levels of *HMGR* normalized using the combination of the top two ranked genes. (D) Relative expression levels of *HMGR* normalized using the least stable gene. The words in horizontal axis represent the callus with different treatments. CK, untreated; ABA, abscisic acid; MeJA, methyl jasmonate; SA, salicylic acid; ACC, 1‐aminocyclopropane‐1‐carboxylic acid; GA_3_, gibberellin A_3_. The error bars represent SD.

Because *HMGR* encodes a key enzyme in the mevalonate pathway in the biosynthesis of terpenes, which has more branches in plants compared with other organisms [39], its expression can be upregulated by a wide variety of external stimuli, such as ABA, MeJA, SA, and other factors [40, 41, 42]. Therefore, *HMGR* expression was selected to validate the candidate reference genes. In the callus group, *HMGR* expression patterns were similar when normalized using *TBP*, *PP2A*, or their combination (Fig. 5A–C), with most hormone treatments promoting *HMGR* expression. However, there were also some slight differences when the selected reference genes were different; for example, when *TBP* was used for normalization, *HMGR* expression was higher in calli treated with ABA than in calli treated with MeJA (Fig. 5A), and there was also a slight difference when *PP2A* was used (Fig. 5B). As a comparison, *bHLH*, the most unstable gene, was used as a reference for normalization; however, this resulted in a different expression pattern. As shown in Fig. 5D, the expression level of *HMGR* was significantly higher at 42 °C than at 4 °C when *bHLH* was used as reference, but the opposite was true when the stable genes *TBP* and *PP2A* were used (Fig. 5A–C).

**Fig. 6:**
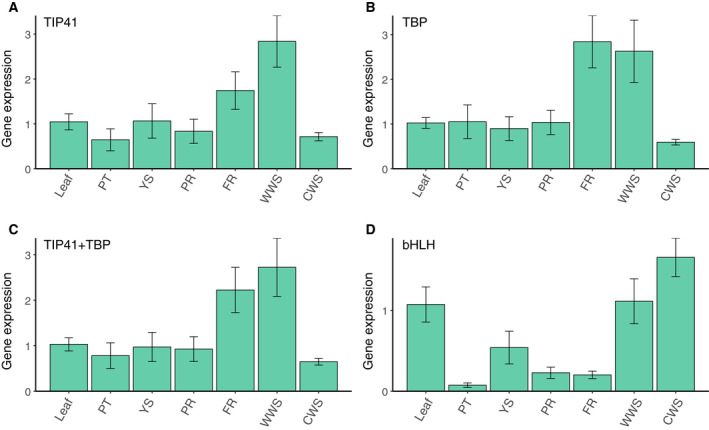
Relative expression levels of *HMGR* normalized using the reference genes in the differentiated group. (A, B) Relative expression levels of *HMGR* normalized using the top two ranked genes. (C) Relative expression levels of *HMGR* normalized using the combination of the top two ranked genes. (D) Relative expression levels of *HMGR* normalized using the least stable gene. PT, petiole; YS, young stem; PR, primary root; FR, fibrous root; WWS, wild woody stem; CWS, cultivated woody stem. The error bars represent SD.

**Fig. 7:**
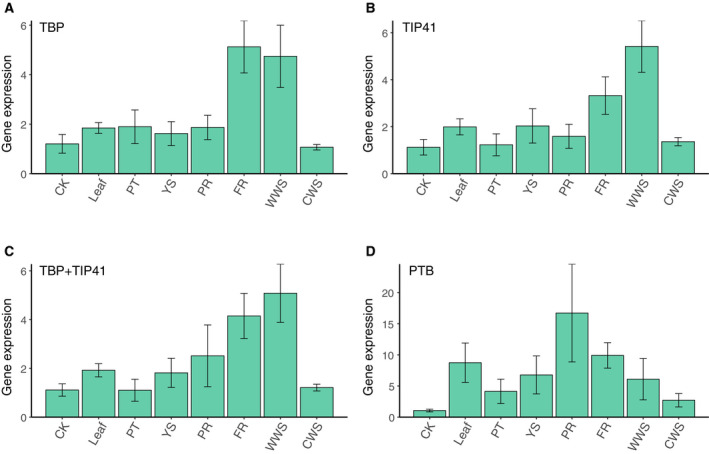
Relative expression levels of *HMGR* using the reference genes in the complete group. (A, B) Relative expression levels of *HMGR* using the top two ranked genes; (C) relative expression levels of *HMGR* using the combination of the top two ranked genes; (D) relative expression levels of *HMGR* using the least stable gene. CK, untreated control callus; PT, petiole; YS, young stem; PR, primary root; FR, fibrous root; WWS, wild woody stem; CWS, cultivated woody stem. The error bars represent SD.

In the differentiated group, *TIP41* and *TBP* were used as the internal controls (Fig. 6A,B); the *HMGR* expression profiles for these genes and their combination were similar (Fig. 6C). When the worst candidate gene was used, the expression data were distributed in a different pattern (Fig. 6D). To evaluate the dependability of the best‐performing genes in the comprehensive ranking for the complete group (Table 2), we tested the expression of *HMGR* in control callus samples from the callus group and other samples from the differentiated group, using *TBP*, *TIP41*, and their combination for normalization (Fig. 7A–C). Calculations using the $2^{‐\Delta \Delta C_{t}}$ method [38] found higher expression of *HMGR* in fibrous root tissue and wild woody stems compared with other samples, and the expression patterns were similar regardless of whether the internal control was *TBP*, *TIP41*, or their combination. However, results were different when the most unstable candidate gene was used for normalization, with the expression of *HMGR* in primary root tissue increasing significantly (Fig. 7D).

## Discussion

The use of reliable reference genes in the normalization of qRT‐PCR data is important for obtaining credible results regarding the expression of target genes. If inappropriate reference genes are selected, highly expressed genes may falsely appear to be expressed at low levels [43]. Although researchers have studied many candidate reference genes in plants [44], there have been few studies on appropriate reference genes in the Oleaceae. Of the studies that have been conducted in this family, one found *EF1α* to be the best reference gene in *Fraxinus* (a genus of trees distributed widely in North America) [45], one reported *UBC2* and *ACT* to be the best reference genes for *Osmanthus fragrans* [46], and one recommended *UBC1* as the most reliable internal control for *Olea europaea* [47]. However, there have been no previous attempts to screen candidate reference genes for expression analysis in the genus *Syringa*. Previous researchers conducting such analyses (working with *Syringa oblata* Lindl. and *Syringa vulgaris* L.) tended to adopt *ACT* as a reference gene [48, 49, 50]. *ACT* is a commonly used reference gene in plants [44]; however, it would not be appropriate for all studies. For example, the expression level of *MAR* in different *Panax ginseng* tissues was much lower using *ACT* for normalization compared to the more stable reference genes screened [51]. Therefore, more reliable reference genes are needed to improve the accuracy of normalization.

The callus group in this study included various *S. pinnatifolia* callus samples treated using different temperatures, salinities, and osmotic pressures, as well as a series of plant hormones; the differentiated group included tissues from various parts of the plant, some of which were taken from plants in different stages of development. Boxplots of the interquartile range (Fig. 2A,B) showed the variation in expression of most genes to be lower in the callus group than in the tissue group. Thus, it is possible that the impact of external treatments on gene expression may be weaker than that of tissue factors.

The geNorm program found that using a combination of three reference genes was the most appropriate normalization method for the complete group (Fig. 4); this is line with previous studies showing that the expression of a combination of genes tends to be more stable than that of a single gene [34]. According to the comprehensive ranking based on the geometric mean of the algorithm‐calculated rankings (Fig. 3, Table 2), *TBP* and *PP2A* were the most suitable reference genes in the callus group, while *TBP* and *TIP41* were the most appropriate for differentiated tissues and for the complete group.

To validate the genes that topped the comprehensive ranking (Table 2), the expression of *HMGR*, which is crucial in the biosynthesis of terpenes, was analyzed in the various samples. When the selected genes and their combinations were used as internal controls, *HMGR* was found to be upregulated in the samples treated with various hormones (Fig. 5A–C); these results correspond to those of previous reports [40, 41, 42], supporting the validity of the selected reference genes. In addition, *HMGR* transcripts accumulated at a higher level in calli treated at 4 °C than in other calli, which was similar to the results of a previous study [52]. In that study, researchers observed that rubber production in *Parthenium argentatum* increased during the cold season in the desert; they found that low temperature promoted the expression of *HMGR*, which is also important in the biosynthesis of rubber [52]. Similarly, wild *S. pinnatifolia* grows at high altitude in a harsh environment exposed to low temperatures. As *HMGR* was distinctly upregulated in our samples after exposure to low temperatures among our samples, it might be worthwhile to explore the relationship between temperature stress and the accumulation of terpenes in *S. pinnatifolia*.

*HMGR* expression in differentiated tissues was highest in wild woody stems and fibrous root tissue (Fig. 6A–C). Previous studies found terpenes to be the major pharmacologically active substances in *shanchenxiang* [7, 8, 16], and our study found that the key enzyme of terpene biosynthesis was significantly upregulated in wild woody stems compared with cultivated woody stems; these results agree with the observation that the best *shanchenxiang* for medicinal use is collected from mature wild *S. pinnatifolia* plants, while the cultivated plants are less medicinally useful. Furthermore, *HMGR* was highly expressed in fibrous root tissue, which might be related to the environment in which such tissue develops; the fibrous roots are buried in soil, exposing them to a greater range of microorganisms and external stresses. Therefore, the fibrous roots may have pharmaceutical potential as a new source of *shanchenxiang*.

In conclusion, this study evaluated the stability of 12 candidate reference genes in *S. pinnatifolia*. *TBP* and *PP2A* were found to be appropriate as internal controls for calli exposed to different treatments. In differentiated tissues, *TBP* and *TIP41* were the best reference genes for data normalization, and the same was true when calli and differentiated tissues were considered as a whole. This study is the first to use reference genes to normalize gene expression data in *S. pinnatifolia*, and it is also the first to screen candidate reference genes in the genus *Syringa*. It will serve as a useful reference and foundation for further research on the molecular biology of *S. pinnatifolia* and other plants belonging to *Syringa*.

## Conflict of interest

The authors declare no conflict of interest.

## Author contributions

JG wrote the manuscript and analyzed the data. JG and JL performed the experiment. CJ validated and supervised the data. SC provided the resources. LH and JL acquired the funding, designed the project, and contributed to the review and editing of the manuscript. All authors have read and agreed to the published version of the manuscript.

## Supporting information


**Fig. S1.** Specificity of the PCR products. A. Specificity of the PCR products and amplicon size of primer pairs. B. Melt peaks of the 12 candidate reference genes.

**Table S1.** cDNA sequences of 12 candidate reference genes.

**Data S1.** The Ct values of 12 candidate reference genes.

## Data Availability

All data generated or analyzed during this study are included in this published article.
