# Supplementary figures and images for: Identification of suitable reference genes for studies of Syringa pinnatifolia Hemsl
Source: FEBS Open Bio. 2021 Feb 26;11(4):1041–53. doi: 10.1002/2211-5463.13097 (PMC8016119; doi:10.1002/2211-5463.13097)

**A**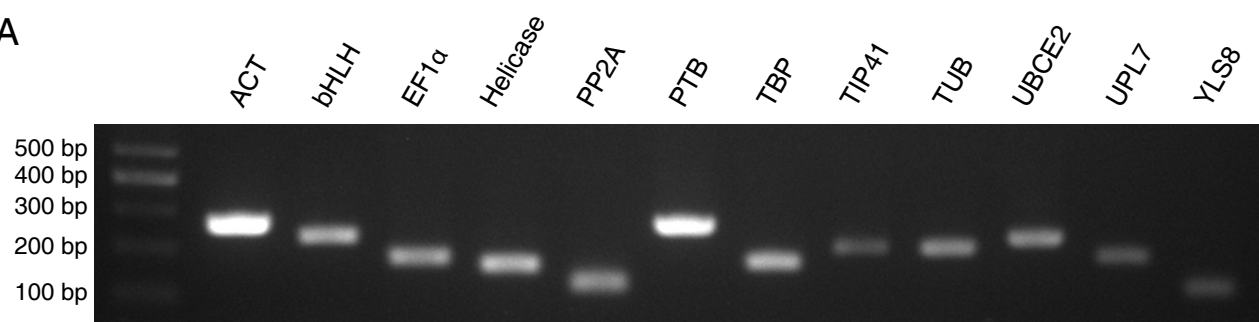**B**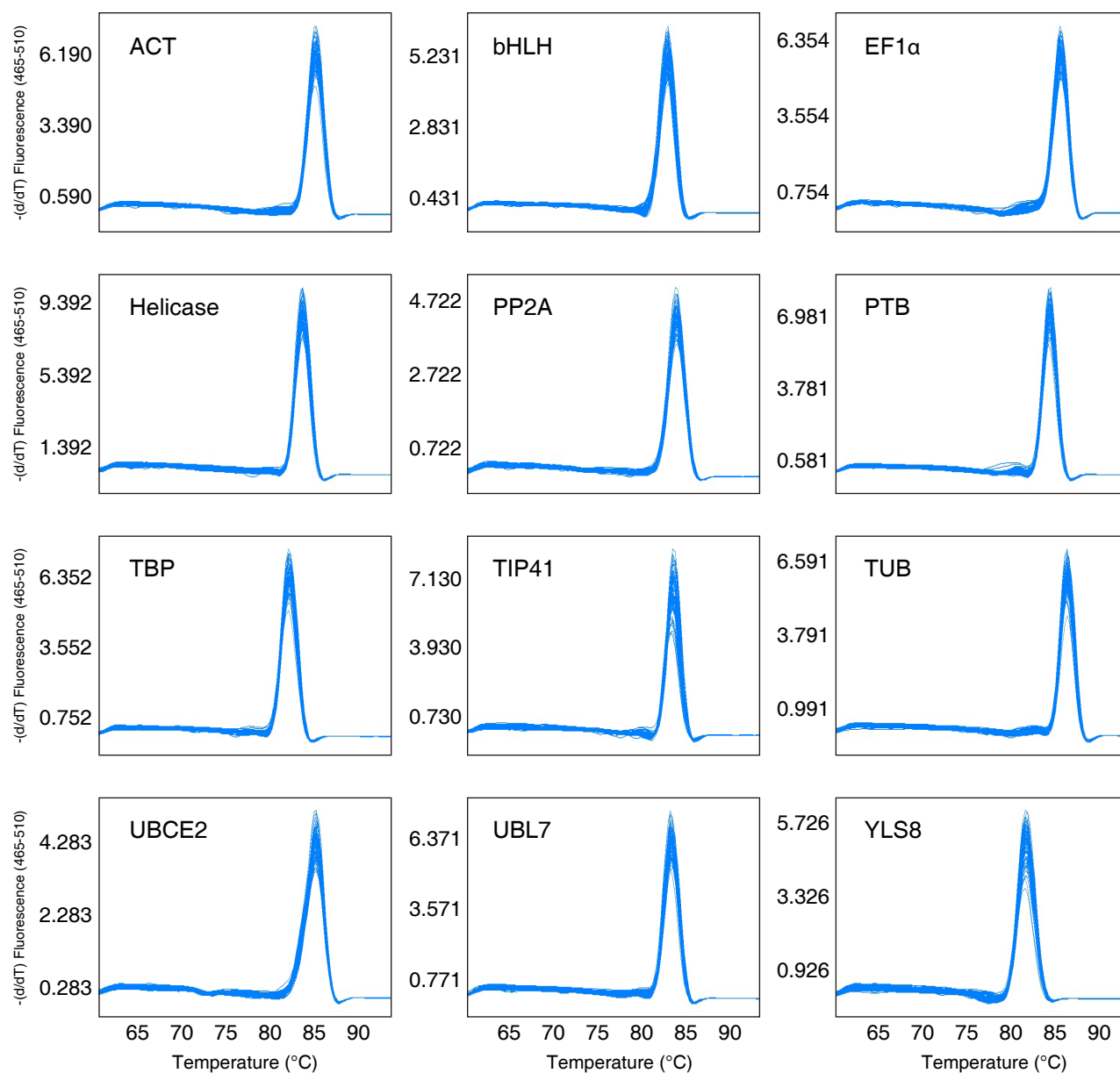

Supplement: Supplementary file 1 — Fig. S1. Specificity of the PCR products. A. Specificity of the PCR products and amplicon size of primer pairs. B. Melt peaks of the 12 candidate reference genes. [file FEB4-11-1041-s001.pdf]
