# Supplementary material for: Identification of suitable reference genes for studies of Syringa pinnatifolia Hemsl
Source: FEBS Open Bio. 2021 Feb 26;11(4):1041–53. doi: 10.1002/2211-5463.13097 (PMC8016119; doi:10.1002/2211-5463.13097)
Supplement: Supplementary file 2 — Table S1. cDNA sequences of 12 candidate reference genes. [file FEB4-11-1041-s002.docx]

**Table S1**. cDNA sequences of 12 candidate reference genes.

>*ACT*

AGAGCATATTAAGAGATGGCAGAAGCTGAGGATATTCAGCCTCTTGTATGTGACAATGGAACAGGAATGGTCAAGGCTGGATTTGCTGGAGATGATGCTCCAAGGGCTGTCTTCCCCAGTATCGTGGGGCGCCCTCGTCACACTGGTGTCATGGTTGGTATGGGCCAAAAAGATGCCTATGTTGGTGATGAGGCTCAATCCAAGAGAGGTATTTTGACTCTAAAATATCCGATTGAGCACGGAATTGTCAGCAATTGGGATGACATGGAGAAGATTTGGCATCATACCTTCTACAATGAACTTCGTGTGGCTCCAGAAGAGCATCCTATTCTATTGACAGAAGCTCCTCTTAACCCCAAGGCAAATCGTGAAAAAATGACTCAAATCATGTTTGAGACTTTCAACACTCCTGCTATGTATGTTGCCATTCAAGCTGTTCTTTCCCTTTATGCCAGTGGTCGTACAACTGGAATCGTTCTGGACTCTGGGGATGGTGTCAGCCATACAGTCCCCATCTATGAAGGTTATGCACTCCCCCATGCAATCTTGCGTCTTGATCTAGCCGGGCGTGATCTCACTGATGCCCTCATGAAGATACTGACCGAGCGAGGCTACTCTTTCACTACATCTGCTGAGCGTGAAATTGTAAGAGATATAAAAGAGAAACTAGCTTACATTGCTCTCGACTATGAACAAGAGCTAGAAACTGCAAAAACCAGCTCATCTGTGGAGAAGAACTATGAACTGCCTGATGGACAGGTGATCACTATTGGTGCCGAGCGATTTCGCTGCCCTGAAGTACTTTTCCAGCCATCAATGATTGGTATGGAAGCTGCTGGCATACATGAAACTACTTATAACTCTATCATGAAGTGCGACGTTGATATTAGGAAAGATCTGTACGGAAACATTGTCCTCAGTGGTGGTTCGACTATGTTCGGAGGAATTGCTGACAGGATGAGCAAGGAGATTACAGCATTAGCTCCAAGCAGCATGAAGATTAAGGTGGTGGCTCCACCTGAGAGGAAGTACAGTGTCTGGATTGGAGGTTCCATTTTAGCCTCTCTCAGCACATTCCAGCAGATGTGGATAGCAAAGGCAGAGTACGATGAGTCTGGACCTTCAATTGTCCACAGAAAATGCTTCTAAAACAACATATGATGAAAAGAGTTCGTTGAGGAATTTATACATTCTATTTTGGAAAAGGCTTTTCATTAAAATTGCTGCTTCTCATTTCATTTTTATTCTTGTTATTTCCTGTGGTCTTTAGCATTCAAGATTTGAATTATTGCAACGAAACGATATCATAGTACCCAATATTCGTTTAGCCATTGTTACCAAATTACAGTATAGTTGAGCATTTTAAATATTATTATGAATTCTGTTTTGGATTAA

>*bHLH*

GCAAGCCAGCCAATGGAAGAGAAGAGCCCTTGAATCACGTAGAGGCAGAGAGGCAGCGAAGAGAGAAGCTGAACCGTCAGTTCTGTGCATTACGCGCAGTTGTTCCCAACATTTCCAAGATGGACAAAGCCTCGCTCTTGGGAGATGCCATCACTTACATAACCCAGCTTCAAAAGAAGGTGAAGGACATGGAGTCTGACGTAGAAAACCACGAAAGCATTTCGAGAAATGCATCAAATTCAGGATTACAAAAAAATCAAGTTGTTAAAATCGACGTCCAATCTGTCCACGACAAAGTCATTGTGAGGGTACTTTGTCCATTGGATGAGCATCCTGTATCAAGGGTCATCCAGGCATTCCAGGATGCACAAGTAACTATTCTAGAGTCGAAGATTGCCACAGGGACTGATGCTGTGTTTCACACATTTGTCGTGAAGTCGCAAGGATCCGAGCAGTTAACTAAGGAAAAGTTGATACAAGTATTTACCCGAGAGTCTAATTCCTTAGAGACTGCTGTATCCTCAATTGCTCGATGATCTCATAATATTACTGCACTTGTTGTGGCATAGTTAATAGGCAAAATGTGTAGTGAGAAATATGGAAAGCTTCTGTAGAGATTTTGATATACATCAGTTGATGCCAGGATTAGGAAAAGTACTATGCTTTGACCTAGTATAAAATCAATTTTCTGTTTGTTTATAGTATACATGATCTGTCATAAGGATGTTTATATGCAATTTATATAAGTTTAACTTGTTTTGCCAGTGTTACTATT

> *EF1α*

TATATAAACCACTTATCCCTTTTCGTTTCACTCTCACTCTTCACAGAAATCAAAATTCTCTCTGCGGTCGGCGGTTCAGAGATTTCTCTATACTACGTTCAAAGCTTTCTTTTTAGAAGTAAAACATGGGTAAGGAAAAGATTCACATTAGCATTGTGGTCATTGGCCACGTCGACTCTGGAAAGTCCACCACCACTGGCCATTTGATCTACAAGCTTGGTGGTATTGACAAGCGTGTCATTGAAAGGTTTGAGAAGGAGGCTGCTGAGATGAACAAGAGGTCATTCAAGTATGCCTGGGTGCTTGACAAGCTCAAGGCTGAGCGTGAACGTGGTATCACCATTGATATTGCCTTGTGGAAATTTGAGACCACCAAATATTACTGCACGGTTATTGATGCTCCTGGACATCGTGATTTTATTAAGAACATGATTACTGGTACTTCTCAGGCTGACTGTGCCGTCCTTATTATTGACTCTACCACTGGTGGTTTTGAAGCTGGTATTTCTAAGGATGGTCAGACCCGTGAGCATGCTTTGCTTGCCTTCACCCTTGGTGTCAAGCAAATGATTTGCTGCTGCAACAAGATGGATGCCACCACACCCAAATACTCCAAGTCTAGGTATGATGAAATTGTGAAGGAAGTGTCATCCTACATCAAGAAGGTTGGATACAACCCTGACAAGATCCCGTTTGTTCCCATCTCTGGTTTTGAGGGTGACAACATGATTGAGAGGTCCACCAACCTTGATTGGTACAAGGGACCAACCCTCCTCGACGCTCTTGACCAGATTCAGGAACCCAAGAGGCCCACAGACAAGCCTCTCCGTCTCCCACTTCAGGATGTCTACAAGATTGGTGGTATTGGAACTGTCCCTGTTGGCAGGGTGGAGACTGGTGTTATCAAGCCTGGTATGGTTGTTACCTTTGGCCCAACTGGTTTGACAACTGAAGTCAAGTCTGTTGAGATGCACCACGAGGCCCTCCAGGAGGCTCTTCCTGGTGATAATGTTGGGTTCAACGTCAAGAACGTTGCCGTGAAGGATCTCAAGCGTGGTTACGTTGCCTCTAACTCCAAGGATGACCCTGCCAAGGGTGCTGCCAGCTTCACATCCCAGGTCATTATCATGAACCACCCTGGTCAGATCGGAAATGGATATGCCCCCGTGCTTGACTGCCACACATCCCACATTGCTGTTAAGTTTGCCGAGCTTCAGACCAAGATTGACAGGCGTTCTGGCAAGGAGCTGGAGAAGGAGCCCAAGTTCTTGAAGAATGGTGATGCAGGTCTTGTTAAGATGATTCCCACCAAGCCCATGGTTGTGGAGACTTTCGCCGAGTACCCACCTCTTGGTCGTTTTGCTGTCAGAGATATGCGACAGACAGTTGCTGTCGGTGTCATCAAGAGCGTGGACAAGAAGGATCCATCTGGTGCCAAGGTCACCAAAGCTGCTGCTAAGAAGGGTGCCAAATGAAGAAAATTCCGGTCGCGTGATATGTTTTTTATTAATCAAATTAGTTATTTTCAAAGACATCTTTTGCAGTTTTGATTATTATGGTTTTAACAGATGCTCTATCTGAATTGCCATTTCTTGTTTTGGTTTTATTGCTTGAGATGAGTTGTAGGCATTCGAACAAATATTCTATTCTGTTTT

> *Helicase*

AGGTTCTGTTGGAGTTATTTCAACTGCACGTGATGATTGGGTTTCTTCAAGAGATCCTTCAGCAATTATAGATGAGGATAAAAAGCCAGAAGTAGCTACCAAAATTATGGTCCGGAAGGAGGACAAACCTCTAGATTCTTTTCAGCGTTCTCAAGCAGATTGGATTCGCCAATACATGGAACAACAGGAAGAGGATGAATTTGATTCTTGGGAAGCTAATCCAATTGACAATAATTCATCGAAGAAGGTCTTGCAACCAAGGACTAATCCCGAGTCTATTGTTGATGAATACCATTCTGCAAGGCTGGAGGCTTTAAATGCCAAAGAGAGAGGAGACAAGAAAGGGCAGGAACAAGCAGGCCTGATCATTAGGAAACTTAAGCAGGAAATATCTGCATTAGGACTATCAGATGATTTCTTGGCATCTGGATATGTAAGTTCTTCCTATCATGCATCTATGAACACGTCTTTCGAGTCCAAGCCTTCTGGAAACTTCGGCAGTGGTACAGTTACTACAAATGATATAGAAGGTTCCACTGCTTCTACTCAGTTTGTGGCTGAAGTTGATCAAAATGTGGAGGATAGCTCTGGCTCACATTTGTGTTCAACCGATAATGTTTCATCAAGTGTTCCCAAAAATGATGGAACTGCTTTGGAAGGGAAATCAGGAGATGTAGAACTTGGTGGTTTCTTCTTTGAAGATGATACCTCAGGAGAAGTTCCTCTGGAAGTATTGGAACTACAAAAGAAAGAAAAATTGAAAGAACTTTCTAGCGGGAAGAATTTAAAGAAACTGGAAGGTATCTGGAAGAAGGGGGATATGCCAAAGGTTCCAAAGGCATTTCTTCACCAACTTTGCCAAAGATCAGGGTGGGAAGCTCCGAAATACAATAAAGTTCTTGGTAGAGATCACAGTTCAGGTTACTCTGTAAGCATATTGCAGAAAGCTAGTGGAAGGGGCAAGAGCAGAAAAGCTGGAGGGCTAACGACTATCCAGCTTCCCGACCAGGACGAAACCTTTAATACTGCTGAGGATGCGCAGAATAAAGTTGCAGCTTATGCACTCCATTGCCTATTTTCAGATCTACCTGTTCACTTTGCACTTTCAGAGCCATACGCTTCTCTTGTGCTGAAATGGAAGGAAGCGGAGTTATTTACTAATGTAAAAGACAATCAGAAAGATCGCAGGGCTGGTTTTGTGGATTCATTATTGAGTGCTGAAAGAATTAAATCAAATGTTCTGTCTGATATCATGGATAGTCCATTTCAAGAGAAGTTTCAAAACCCATATATTGCAGAAGACATTACTGGTGGCACCAATCAAAATGCTGAAAGAATAAACAAGGATAAACATGCAGAGAGCTTCTATCTGAAAAAGGAACAGGAGCGTAAGAAAGGAATGAAGAAGTGCAAGGAGGAGATGTTGGAGTCCAGATCCAGACTCCCTATTGCAGAGTTGAAAGGTGATATTCTGCATTTGCTTGAGGAGAATAATATTTTGGTTATATGTGGGGAGACAGGCTGTGGAAAAACAACTCAGGTCCCACAATTTATATTGGATAACATGATTGAAGCAGGACGTGGTGGGTTCTGTAACATAATATGTACTCAACCCAGGAGAATTGCGGCCACGTCTGTCTCTGAAAGAGTTGCTGATGAGCGATGTGAACCGTCTCCAGGATCAAATGGTTCGTTGGTTGGTTATCAAGTTCGTCTCGATAGTGCAAGGAATGAGAGGACGAAACTTCTTTTCTGTACAACTGGCATTCTCCTGAGGATGATATCGGGGAATAAAGACTTGGCTGGTGTTAGTCATGTTATTGTTGATGAAGTGCATGAACGCTCTCTTTTGGGGGACTTTCTGCTCATTGTTATGAAGAATCTGATTGAAAAGCAATCTACTCACAGCAAATCTAAGTTGAAAGTTATTCTTATGTCTGCAACAGTTGACTCACACATGTTCTCACAGTACTTTGGTAATTGCCCAGTAATTACTGCTCAAGGGCGAACACATCCCGTGTCAATCCATTATCTTGAGGATATATATGAAACCTTGGAATACCGTCTTGATTCAGATTCTCCTGCCTGTATAAATCATGGGATATCTGTGTCTGAGAAGAGTGCTCCTGTTGGCAACCGGAGAGGAAAGAAAAATCTTATCTTGGCGTCTTGGGGTGACGAGTCCATACTGTCTGAGGAGTGTATCAATCCATTTTATGATTCAAGTAATTATCAAAATTACAGTGAGCAAACTCGCCAAAATTTGAGAAGATTGAATGAAGATATTATTGATTATGAACTTCTTGAGGATCTGGTATGCCATATTGATGAAACTCATGCTGAGGGAGCTATATTAGTCTTTTTGCCGGGAGTTGCAGAAATAAATATGTTACTGGATAAACTTTCTGCTTGTCACCAATTTGGTGGATTGGCCTCTGAATGGCTGCTTCCTTTACATTCATCTATAGCATCTGAAGATCAAAAGAAAGTGTTTCAGAAACCTCCTGATAACATTCGTAAGGTTATAATTGCCACAAATATTGCAGAGACAAGTATAACCATAGATGACGTGGTATATGTGATAGACTGTGGTAAGCACAAGGAGAATCGTTATAATCCACATAAGAAACTATCAAGCATGGTTGAAGATTGGATATCTCAAGCAAATGCAAGGCAACGGCGAGGAAGAGCTGGACGTGTGAAGCCTGGAATCTGCTTTTGTTTGTATACACGCCACAGATATGAAAACCTCATGCGTCCATATCAGACGCCTGAGATGATGCGGATGCCATTGGTAGAGTTGTGTTTGCAAGTGAAGCTGCTTTCTCTTGGTGGCATAAAGCAGTTTTTGTCTAAGGCTCTGGAACCTCCAAAAGAAGAAGCTATAGAATCAGCAATTTGTTCATTATACGAGGTTGGTGCCATCGAGGGAGATGAAGAGCTGACACCTCTTGGATATCATTTGGCTAAACTCCCAGTCGATGTATTAATTGGAAAGATGATGTTATATGGTGGAATATTTGGTTGTTTGTCACCAATTCTCACTATTTCAGCGTTTTTGAGCTACAAGTCGCCATTTGTTTATCCAAAAGATGAGAGGGAAAATGTTGAAAGAGCGAAGTTGGCCCTGGCTACTGATAAGATTGGTGATGCAACAGTTCAGATTGATGCTATACGGCAATCTGACCATCTTGTAATGATGATGGCATATCAGAAATGGGATAAAATTGTGCATGAGCATGGAATAAGAGCAGCACAAAAATTTTGCAGTTCACATTTCCTGAGCAGCTCTGTCATGTTTATGATAAGGGATATGAGAATACAATTGGGTACATTGCTTGCTGACATTGGGATAATTAATATTCCGAAAAATTATCAGGTTGGATGGAAAAAGAAAGAAAAGCTTGACAATTGGCTTTCAGATTCATCACAACCATTCAATCGGTATTCATGTCATTTTTCAGCAGTGAAGGCAATATTATGTGCAGGTCTGTATCCTAATGTAGCCATGATTGAGGAAGGCAGTCCTGAGGGTCGTCCTGTTTGGTACGATGGAAAAAGAGAAGTTCACATACACCCTTCTTCTGTTAATGGTAACCAGAGAGCTTTTCAGTATCCATTTCTCGTCTTCCTCGAAAAGGTTGAGACGACTAAAGTATTTTTACGAGACACTACCGTGGTTTCCCCCTACTCTATTTTGTTGTTTGGTGGACCAATGAATGTTCACCATAAGACGGGACTAATTGCTGTAGATGGTTGGCTAAAAATGGAAGCACCAGCACAAACTGCTGTCCTCTTTAAAGAACTTAGATTGACTCTTCATTCTATTCTGAAGGAGCTCATTCGTAATCCACAG

>*PP2A*

ACAAATCTGTGGAGTCATTGTGTAGAATTGGATTACAGATGAGGGAGAATGATATGGCCGAAAGGTTTATCCCTCTTGTGAAGAGGCTGGCAGCTGGTGAGTGGTTTACAGCTCGAGTTTCTGCTTGTGGCCTGTTTAATGTTGCTTACCCCAGTGCCCCAGATGTGTTAAAGACAGAGCTAAGGTCAATATACAGCCAACTGTGTCAAGATGATATGCCAATGGTGAGAAGGGCTGCTGCAACTAATTTGGGGAAATTTGCCGCTACAGTAGAAGCTTCTCATCTCAAGACTGATATCATGTCAATGTTTGAGGATCTTACTCAGGATGATCAAGATTCTGTTCGCTTGCTAGCTGTTGAGGGCTGTGCGGCGCTTGGCAAGTTGTTGGAGCCTCAAGATTGTGTTGCACATATCCTCCCTGTAATTGTCAATTTTTCGCAGGATAAGTCTTGGAGGGTTCGCTACATGGTTGCAAACCAGTTGTATGAGCTTTGTGAAGCTGCGGGACCTGAATCTACTAGGAGGATGGATTTGGTTCCTGCTTATGTGCGCCTTCTTCGAGATAATGAAGCTGAAGTACGCATAGCTGCTGCTGGGAAAGTCACCAAATTCTGTCGGATTCTTAATCCTGAACTTTCTATTCAGCATATTCTTCCCTGTGTGAAGGAATTGTCATCGGATTCTTCACAACATGTTAGGTCTTCTTTGGCTTCTGTTATAATGGGAATGGCTCCCGTGTTAGGGAAGGATGCAACAATTGAACAACTTCTTCCAATATTTCTTTCCCTTTTGAAGGATGAATTTCCTGATGTACGCCTCAATATTATTAGCAAACTGGATCAAGTAAATCAGGTTATTGGAATTGATTTGCTATCCCAATCTTTATTGCCAGCTATTGTTGAGCTAGCAGAGGATAAGCATTGGAGGGTTCGCCTTGCGATTATAGAGTATATACCTTTATTGGCTAGTCAATTGGGTGTAGGATTTTTTGATGATAAGCTTGGTGCCCTTTGTATGCAATGGTTACAGGACAAGGTTTACTCTATTCGAGATGCAGCTGCTAATAATTTGAAACGACTTGCGGAAGAATTTGGTCCAGATTGGGCAATGCAGCATATAGTTCCTCAGGTTTTGGATATGATAAAAAATCCGCATTATTTGTACCGGATGACTGTTCTTAGTGCAATTTCATTACTTGCCCCTGTCATGGGTCCTGAGATAACACGTTCTCAGCTGTTGCCTGTGGTTGCTACGGCATCAAAAGACAGAGTTCCCAACATTAAATTTAATGTGGCCAAGGTGTTGCAGTCCCTTATTCCCATAGTTGATCAACCGGTGGTGGAGACAACCATCCGACCCTGTTTGGTGGAGCTCACCGAGGATCCTGATGTTGATGTCCGGTTTTTTGCCAATCAAGCACTTCAGTCAATTGATAATGTCATGATGTCGAGCTAGACGAGCTCCTTCTGTGGAAAGTTCATTTCTGGAATTTTGTTTTGAATATCCTTTTCATTTGGTTCTTCAACAACCTAGTTTGGCTGAAAAAACCTTCTTGTTATGAT

>*PTB*

GATGTCGACGTCGGGGCAGCAGCAATTCCGGTACACGCAAACTCCGTCGAAGGTTCTCCACCTGCGGAATCTCCCGTGGGAGTGCACCGAAGAAGAGCTGGTCGAGCTCTGTAAACCTTTCGGCAAGATCGTCAATACCAAGTGCAATGTCGGCGCCAATCGCAACCAAGCTTTTGTTGAACTCGTGGATCTTAATCAGGCAATTAACATGGTTACATATTATGCTTCGTCTTCCGAACCTGCAAATGTACGTGGCAAAACTGTCTATATCCAGTACTCGAACAGGCATGAAATTGTCAACAACAAGAGTCCTGGAGATGTTCCAGGCAACGTCTTGCTTGTTACAATCGAGGGAGTTGAAGCTGGTGATGTCAGCATTGATGTCATTCACTTGGT

>*TBP*

GAATATTGTGTCGACGGTTAATCTGGAATGCAAATTGGATCTAAAAGCTATTGCATTGCAAGCTCGCAATGCAGAATACAATCCTAAGCGTTTCGCTGCAGTGATTATGAGGATTAGAGAACCAAAAACAACGGCCCTAATATTTGCTTCTGGAAAGATGGTTTGTACTGGAGCCAAGAGTGAACAACAGTCGAAATTGGCTGCTCGGAAGTATGCTAGAATTATTCAAAAGCTCGGCTTCCCAGCAAAATTTAAGGATTTCAAAATTCAGAACATTGTTGGTTCTTGTGATGTTAAATTTCCCATTCGACTTGAAGGCCTTGCATATGCTCATGGTGCCTTTTCAAGTTACGAACCTGAATTGTTCCCTGGATTAATCTATCGGATGAAACAACCAAAGATTGTGCTTCTTATTTTTGTCTCCGGAAAGATTGTTCTCACAGGAGCCAAGATCAGAGATGAGACATATGCTGCCTTTGAGAACATATACCCCGTTCTCACTGAGTTTCGGAAGGTTCAGCAATG

>*TIP41*

TTGGGCTTTGACACGACCTTACTAATTCCCAGTTGACCGTCGAAAAATCGGAGGTGTTAGCAAATTATCGGAGAATATGGAATTGGATAGCGACGAAAGGGAGCTGAAGGCCGCCGGAGCTGAACCGCTCGACGTCGGTCGCCTTGGCCTTCGCATTCGTGGGTGGGAAATCGAATCGCGCAAGCTTCCCATACTCAACTCTTCGCTTCTCCAACAGTGGGAACAGAAGCTTCAGACATCCCACTTTCCAGAGATGATATTCGGGGAAAGTTCTTTGGTTCTTAAGCATGTGAACAGTGGCATTAAGATTCATTTTAATGCATTTGATGCTCTCCTTGGTTGGAAGCAGGAGTCATTACCACCAGTGGAAGTTCCTGCAGCGGAACAATGGAAATTCAGAAGCAAACCTGATCAGCAGGTGATACTCGATTATGACTATACCTTCACTACACCATACTCTGGAACCGAAAGTATTGAGACGAATGCTAAGAGCCTGGAAGAGGAGCTCGTTGGGAAGACTGCAATGAGAAAATTGACATGATTGCCTTGGCATCCAAAGAGCCTATTCTTTTCTATGATGAGGTCATCTTGTATGAAGATGAATTAGCTGACAACGGTGTCTCTCTTTTGACAGTTAAAGTGAGAGTCATGCCCAGTGGTTGGTTTCTTCTCTTGCGTTTTTGGCTGAGAGTGGACGGAGTGCTTATGAGATTAAGGGAAACGCGTATGCATTGCGTTTTTGGGGAGACTTCATTGCCCGTTATTCTTCGAGAAATCTGCTGGAGAGAAGCCACATTTCAAGCCTTGTCATTGAAAGGATATCCTACTGATTCTGCTGCTTATAATGATCCTGGCATCATCAGCAAAAGGCTCCCAATCATCATGCACAAGACCCAAAAGCTTTCAATCACATAGATCATGTAAGCTTTAATGAATTCTTAATATTATTTATACTTGAAGAAACCCATAACCAATCTTGTTGGAGAAAAGCAAACGGAGTTATATATAATTGTGAGTATTATTTTTATAATTGCAGAAACCAGGGAAAGAAATAGAAGAAAAAATGTGTTCTTCATATTGTGAATTCCATACGCTAGCATGCAGTTTCTTGTTCTTTCCACTCATGTTTGATTTGTTATAGTGTTGAGTAATCCCATTTTATGAAAGCAAATCTCCAGTGCACTTTTAA

>*TBP*

GAATCGCTTGCCTCTTTAAATAGCCCACTCCCATAATTGTAGCTTCCTCTTAACATTTCCATTAACTGTCCATAAAAATATCAAATATTATTCACAAACAACCAAATCAAAACCCACACAAAATGAGAGAAATCTTGCATATTCAGGGAGGCCAATGCGGGAACCAAATTGGGTCGAAATTCTGGGAAGTAATCTGTGATGAGCACGGGATTGATCCGACAGGTAGGAACAAGGGTGAGGCTGGTGACGGTTCGTCCGATCTGCAGTTGGAGCGGATCAATGTGTATTACAATGAGTCTTCCGGTGGAAGGTATGTTCCGCGAGCTGTTTTGATGGATCTCGAGCCTGGGACCATGGATTCCATCAGATCCGGCCAGTATGGGCAGATCTTCCGACCCGATAACTTTGTTTTTGGGCAGTCTGGGGCTGGGAATAATTGGGCTAAGGGGCATTATACGGAGGGCGCTGAGTTGATTGATGCCGTTCTGGATGTTGTGCGGAAAGAGGCGGAAAATTGTGATTGCTTGCAAGGATTTCAAGTATGTCACTCGCTTGGAGGAGGGACAGGTTCTGGTATGGGTACCCTCTTGATTTCAAAGATAAGAGAGGAATACCCAGACAGAATGATGCTCACTTTCTCTGTTTTTCCATCACCAAAGGTCTCGGACACCGTTGTAGAACCCTATAATGCTACACTTTCAGTGCACCAGTTGGTGGAAAATGCAGATGAATGTATGGTCCTTGACAATGAAGCGCTCTATGAAATCTGTTTCAGGACCTTGAAGCTCAGTAATCCAAGCTTTGGTGATTTGAACCATTTGATCTCTGCAACTATGAGTGGGGTAACCTGTTGCTTGAGATTTCCCGGCCAGCTGAACTCGGATCTCCGGAAGCTAGCTGTGAACTTGATACCTTTCCCGCGTCTTCACTTCTTCATGGTTGGATTTGCGCCACTCACCTCTCGTGGATCACAGCACTACATCTCTCTCACAGTCCCGGAGCTAACTCAACAAATGTGGGACTCGAAGAACATGATGTGCGCTGCTGACCCACGTCATGGACGTTACCTGACAGCCTCCGCCATGTTCAGAGGGAAAATGAGCACCAAAGAGGTCGACGAACAGATGCTTAACGTGCAGAACAAGAACTCATCATACTTCGTCGAATGGATACCAAACAATGTCAAGTCTAGTGTTTGTGACATTCCACCAACTGGCCTGAAAATGGCATCCACTTTCATAGGTAATTCAACTTCGATTCAAGAAATGTTCAGGAGAGTGAGCGAGCAGTTTACGGCCATGTTTCGACGCAAGGCCTTCTTGCACTGGTACACTGGAGAAGGGATGGATGAAATGGAGTTCACTGAAGCTGAGAGTAATATGAATGATCTGGTTGCAGAGTACCAGCAATACCAGGATGCCACTGCAGAGGAAGAAGACGACTACGAGGAGGATGGCGTTGAAGAGCAGTATGAGGGCTAAAAC

>*UBCE2*

ATCAGATTCTACTAATCAACCCAACAAATGAATCAAAACTTTTGCCTCTCTCTGATTTTCCTCTAAAATTGTTTTTACGCCATGAGCTCTACCTCTGCTGCTTCACGCAAGGCTTTGAGCAAGATTGCATGCAATCGGCTACAGAAAGAGCTTACGGAATGGCAGGTGAATCCGCCGTCGGGTTTCAAACACAAAGTAACGGATAATCTGCAGAGGTGGGTAATCGAAGTCAACGGTGCACCGGGGACGCTGTACGCAGGTGAAACGTATCAGCTGCAGGTTGATTTTCCGGAGCATTACCCGATGGAAGCGCCTCAGGTTATATTTATACCTCCAGCCCCACTTCACCCTCACATTTATAGCAATGGGCATATATGTCTAGATATACTGTATGACTCGTGGTCCCCAGCTATGACTGTTAGTTCAATTTGCATCAGCATTCTCTCCATGTTATCGAGCTCGCCTGCCAAGCAACGTCCGGCCGACAATGATCGCTATGTGAAGAATTGCAAGAATGGAAGATCTCCAAAGGAGACGAGATGGTGGTTCCATGATGATAAAGTGTAATAACTTAATTAGCCGAACCCATAGCACCAACTTGTGTATGGGTTGTAAGTGTTGTATTTCTTCCCCTTTGAAAAGAGTAGGGACTGAAAATACTGTAAAAAATGAAAAAAAAATAAAAAAATCTTTTATTTCAATTACTCTAAATGACATGGAGGCATCGAAAGAAACATTTTAAAAAGCTGCTGCGGGCTACTATGCCAAAGTGGGCAGGATTATATTAACTTATAATGAGGTAAATGTGTTTTCCCTTTGCC

>*UPL7*

CCTCGGCAACACCAGGTGTCACTGCGTGGAGCAAGTGCCCGGGAAATATCGAGGGATGCGCTATTGGAGAAAGTTAATCAAGAAAGGGAGCTGCGAAATTATACCAAGCGAGCCACCGCCGCCGCGTCATTTATCCAGAGGGTTTGGAGGCGGCACAATGAGACGAAATTGGTAGCATTGCAGCTGCAGAAGGACTGGGAAATAATGATTAACAAACGGGCTGGTTCTTTTAGCGGAATGCAGATATCGATTGGAGTTTTGAGACCTTTTCTTTTCTTCATTAAGTATTTACCAACATGGCGGGGGGAAAGTCAAGTGGCTGATAGAGATTGTATGATAAATTGCTTCAAACTTGTTTTGGAGAGCATAAATTCATCTGACGGGCATCAAAATTTTTGCTCAATGGCAACTGGTACAGTTGAAGAGAGAAAAACATGGCTTTATCAATCGAAGAAATTGATCTCTGTTTGCATGTCCATTTTAGCAAATTTTGATTACTCCAAACAAGGGGATAAAGATGTTGTTCTTACTTCATTAGCAATGCGTTTAGCAGTTATCTTGACTGATCCGAAAGGTTGGAAAATCATTTCTGATGACAATCGCGAGGATGCTAATACAACAGTAAAAAAACTCGTACAATTTATGGGTAGTACAAGAAGTGGGTTGTACAATTGCATTAGGAAATTCATTTATAAATTGGAAGCTCCCTTTTCTTCTCAAGGGGTCTCTTCTTGCCAGACTGATGATAGATTCTTGATAGTAGCTAGTGCAGTCACTTTATGTTTACGGCCATTCCACATTGCAGACATGGATGTAAATGATGATGGCATGTTGGAGAGTGCTGTCAAACAGTACTGGGTGGTACTACTTACTATACCATGGCTTGCTCAACGTTTACCCTCAATACTTTTACCTGCTCTGAAACACAAGTCCGTATTGTCACCTTGCTATAGGATGCTACTGATTTCAAAAGAGAAGATTCTGGAGGAGATTTCTGAGATGGATAGGTCAAAAATAACTTCTCACTGTAAACTGATGCCACAAGTTGGCTGGGCTCTAGCAAACGTGGTATGCCTTGCAACATGGTGTGACACCAGTGCTCTAGATTCAGGAAAATTCACGCCGGGGTTAGACTATGCATCTTATCTGCACGTTGTTATAATACTCGCAGAGAAACTATTGGATTCACTCGAAAATTTTGGATGGTTTATGAAGGAGGATGAAGATATCCTAGCCGATAGTAATACTTCTGCTTCTTTTGAACTGTTGAATGAGGCTGAAATAACTTATGGGTCCTCAAAAATGTTATACATGGACCTTCTTAAACCTGTTTGTCAACAGTGGCATCTTAAGAAATTGTTATCTTTGTTTAAAGATTCGTCTATACTGGAAACTAATAATCTGCCATCAGGCGATCCGGATAATTTATGGAAGCGTGAATTGCTCAACATAGCTAATTATTATTCATGCATGCTTCGATTATTTTCAACTTTGAATCCTGTTTTTAAATCGTTGCCTATTCTAAATATGCTGTCTTTTACCCCTGGATTCCTTTTCAATTTGTGGGAAGTGCTAGAAAAATCCCTTTTTCCTGAAAAAAGTCATATTGCTAAGGGATACTCGAACCATGGAAATAATATCTATGAGGACAAAAGGGATGGAATTTCTGAGAGAAGTCAAAAGAGATTTGGTGGAGATGGAGGTAATAAATGGGTTAATGTGTTGCATAAATTCACTGGAAAGTCACAAGCAGAAAATGATCGAACGGAATCAAGCAAAATATTATCCAGTTTTAACCAAGTTGATGAACATTCTTCCGATGAATGGGACATAGAGCTGCTAAGACAGGGTCCTGATGGTATATCGAAAGATATGTCTTGTCTACTTCATCTCTTCTGCTCCACTTATTCACACCTGTTATTGGTTCTTGATGACATAGAGTTTTATGATAAACAGGCTCCCTTCACATTAGAGCAGCAGCGGAAAGTTGCCTCAATGGTAAATACACTTGTATATAATGCCTTTTCCCAGAGTATCAACCCACAGAACAAACCTCTCGTGGATTCTGCAACTCGATGCCTGCATTTACTATATGAAAGGGATTGCAGGCACCAGTTTTGCCCTCCTACTTTGTGGCTTTCACCTGGTAAAAATAGTAGGCCGCCGATTGCTGTTGCTGCCAGGACTCATGGGGTTCTGTCAACTGCAGATGGAACACCCTCTTCAAGCATGGGTTCTGTCATAACTACGACCCCACACATCTTCCCATTTGAGGAAAGAGTTAAAATGTTCAGAGAATTTATCAACATGGACAAAGCAACTAGAAGAATGGCCGGTGAGGTCGCAGGGCCTGGTTCACGATCAATTGAGATAGTTATTCGCCGTGGTCATGTTGTTGAAGATGGATTCCGACAATTGAATGCTCTTAGGTCCAGGCTGAAGTCCAGTATCCATGTTTCATTTGTGAGTGAATCTGGCCTCCCAGAGGCTGGCCTTGACTATGGCGGGTTGTCCAAAGAGTTCCTGACTGATATCTCAAAGGCAGGCTTCTCGCCAGAGTATGGGCTTTTCTCCCAGAGCTCAACTTCTGACAGACTTCTAATCCCTAATACTGCTGCTAGATATTTGGAGAATGGTATCCAGATGATTGAGTTTCTTGGAAGAATAGTTGGCAAAGCACTCTATGAAGGAATACTGCTTGATTTTTGTTTTTCCCATGTTTTTGTTCAAAAGTTGTTGGGCCGCTATAGCTTTCTTGATGAACTATCAACCCTTGATCCTGAGCTCTACAAGAATCTTATATATGTTAAG

>*YSL8*

GCTGAAACATTAAAGATCTTTGCTGTTATTTACTTGGTGGATATCACGGAGGTGCTGGATTTTAACACAATGTACGAGTTATATGACCCATCCACTATCATGTTCTTCTTTAGAAACAAGCACATTATGATTGATCTTGGCACTGGAAACAACAACAAGATCAATTGGGCACTGAAGGATAAACAGGAGTTCATCGACATTGTCGAGACGGTTTACCGTGGTGCAAGGAAAGGCCGTGGTCTAGTTATTGCTCCGAAAGATTACTCCACTAAATAGCGTCACTGAGTTCTCATCTAAATCTTACTATTTATGCAGTATAGTATCTTACAATCTTCTTGTATGTTGATTTGGTGAAAACCTTCACAATTTCAGTTCCTATATGGAACTGGTTTCTCAAGCTTCTATGCTAGTTGAAGGTATGAAAGATTTACGCAGCAATTAATGTCTGGTTTGTGGTGTATATCTGATGGCCCTTTGGACTTCATCAATGTATTCAGATTTGAGGATTGTTATCAAGTGGATTGCATCTTTAGATGCGACTCGTGAGCTCTGCTTCACGTTCTTTTTATATGTTTTATATTCAAATTTTTAGATGGTTATCGTGACATCAGAAGTACTTTTCTATTAAATGATGTCTGAATTCTTCAATGCTA
